# (*E*,*E*,*E*)-1,6-Bis(4-chloro­phen­yl)hexa-1,3,5-triene

**DOI:** 10.1107/S160053681300932X

**Published:** 2013-04-13

**Authors:** Zhiying Li

**Affiliations:** a Departmemt of Chemistry, Xinzhou Teachers University, Xinzhou, Shanxi 030006, People’s Republic of China

## Abstract

The title mol­ecule, C_18_H_14_Cl_2_, lies about an inversion centre. The hexa­triene chain is planar with a maximum deviation of 0.0001 (17) Å. The torsion angle of the single bond between the chain and the benzene ring is −168.49 (17)°. In the crystal, the shortest inter­molecular distance between the Cl atoms is 4.0785 (11) Å.

## Related literature

For the preparation, see: Spangler *et al.* (1989 ▶). For luminescence and fluorescent properties of *trans*-diphenyl polyenes, see: Alford & Palmer (1986 ▶); Sonoda *et al.*(2003 ▶).
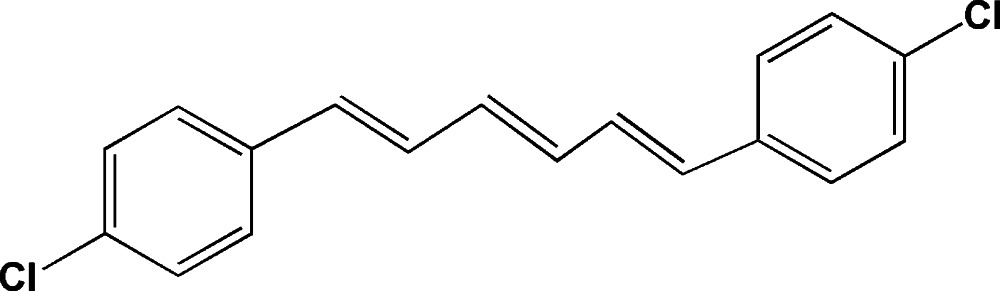


## Experimental

### 

#### Crystal data

C_18_H_14_Cl_2_
*M* *_r_* = 301.19
Monoclinic, 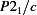

*a* = 15.6277 (7) Å
*b* = 4.0784 (2) Å
*c* = 12.1026 (5) Å
β = 105.810 (4)°
*V* = 742.20 (5) Å^3^
*Z* = 2
Mo *K*α radiation
μ = 0.42 mm^−1^
*T* = 291 K
0.42 × 0.38 × 0.30 mm

#### Data collection

Agilent SuperNova (Dual, Cu at zero, Eos) diffractometer
Absorption correction: multi-scan (*CrysAlis PRO*; Agilent, 2012 ▶) *T*_min_ = 0.667, *T*_max_ = 1.000
2513 measured reflections
1560 independent reflections
1214 reflections with *I* > 2σ(*I*)
*R*_int_ = 0.013

#### Refinement

*R*[*F* ^2^ > 2σ(*F* ^2^)] = 0.039
*wR*(*F* ^2^) = 0.105
*S* = 1.06
1560 reflections
91 parameters
H-atom parameters constrained
Δρ_max_ = 0.17 e Å^−3^
Δρ_min_ = −0.21 e Å^−3^

### 

Data collection: *CrysAlis PRO* (Agilent, 2012 ▶); cell refinement: *CrysAlis PRO*; data reduction: *CrysAlis PRO*; program(s) used to solve structure: *SUPERFLIP* (Palatinus & Chapuis, 2007 ▶); program(s) used to refine structure: *OLEX2* (Dolomanov *et al.*, 2009 ▶) and *SHELXL97* (Sheldrick, 2008 ▶); molecular graphics: *OLEX2*; software used to prepare material for publication: *OLEX2*.

## Supplementary Material

Crystal structure: contains datablock(s) I, global. DOI: 10.1107/S160053681300932X/go2085sup1.cif

Structure factors: contains datablock(s) I. DOI: 10.1107/S160053681300932X/go2085Isup2.hkl

Supplementary material file. DOI: 10.1107/S160053681300932X/go2085Isup3.cml

Additional supplementary materials:  crystallographic information; 3D view; checkCIF report

## Figures and Tables

**Fig. 1.**
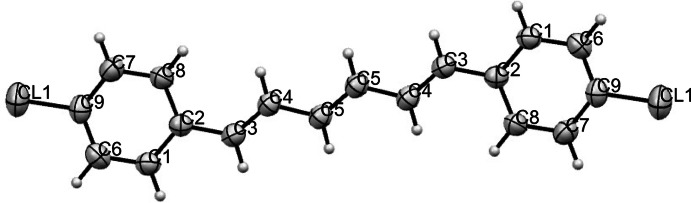
The structure of the title compound in 30% probability ellipsoids. H atoms are shown as small spheres of arbitrary radii.

## supplementary crystallographic information

### Comment

The luminescence properties of the linear all-*trans*-diphenyl polyenes Ph-(CH=CH)_n_—Ph (DPH) have been the subject of numerous investigations(Sonoda *et al.*(2003)). The emission properties of DPH and its derivatives in solution have been extensively studied because of its unique fluorescence behavior(Alford & Palmer,1986). DPH is known to exhibit dual fluorescence from S_1_ and S_2_ at thermal equilibrium. The crystal structure of (*E*,*E*,*E*)-1,6-bis(2,4-dichlorophenyl)hexa-1,3,5-triene has been studied (Sonoda *et al.*2003). In the crystal structure of the related compound, *E*,*E*,*E*-1,6-bis(*p*-chlorophenyl)- 1,3,5-hexatriene, the benzene rings are π···π stacked with unit translation along the *b*-axis with a centroid to centroid distance of 4.0785 (11)Å, a perpendicular distance between the planes of 3.4728 (8)Å and a slippage of 2.139Å.

### Experimental

The compound was prepared and purified as described previously (Spangler *et al.*1989). Crystals suitable for the X-ray diffraction study was obtained free evaporation of an solvent chloroform from a highly diluted solution in the dark at room temperature.

### Refinement

H atoms attached to C atoms were placed in geometrically idealized positions with C*sp*^2^ = 0.930 Å,and constrained to ride on their parent atoms, with *U*_iso_(H) = 1.2*_U_*_eq_.

### Crystal data

**Table:**

C_18_H_14_Cl_2_	*F*(000) = 312
*M**_r_* = 301.19	*D*_x_ = 1.348 Mg m^−^^3^
Monoclinic, *P*2_1_/*c*	Mo *K*α radiation, λ = 0.7107 Å
Hall symbol: -P 2ybc	Cell parameters from 971 reflections
*a* = 15.6277 (7) Å	θ = 3.4–28.6°
*b* = 4.0784 (2) Å	µ = 0.42 mm^−^^1^
*c* = 12.1026 (5) Å	*T* = 291 K
β = 105.810 (4)°	Block, yellow
*V* = 742.20 (5) Å^3^	0.42 × 0.38 × 0.30 mm
*Z* = 2	

### Data collection

**Table:**

Agilent SuperNova (Dual, Cu at zero, Eos) diffractometer	1560 independent reflections
Radiation source: SuperNova (Mo) X-ray Source	1214 reflections with *I* > 2σ(*I*)
Mirror monochromator	*R*_int_ = 0.013
Detector resolution: 16.0733 pixels mm^-1^	θ_max_ = 26.7°, θ_min_ = 3.4°
ω scans	*h* = −11→19
Absorption correction: multi-scan (*CrysAlis PRO*; Agilent, 2012)	*k* = −4→5
*T*_min_ = 0.667, *T*_max_ = 1.000	*l* = −15→15
2513 measured reflections	

### Refinement

**Table:**

Refinement on *F*^2^	Primary atom site location: structure-invariant direct methods
Least-squares matrix: full	Secondary atom site location: difference Fourier map
*R*[*F*^2^ > 2σ(*F*^2^)] = 0.039	Hydrogen site location: inferred from neighbouring sites
*wR*(*F*^2^) = 0.105	H-atom parameters constrained
*S* = 1.06	*w* = 1/[σ^2^(*F*_o_^2^) + (0.0404*P*)^2^ + 0.1061*P*] where *P* = (*F*_o_^2^ + 2*F*_c_^2^)/3
1560 reflections	(Δ/σ)_max_ = 0.001
91 parameters	Δρ_max_ = 0.17 e Å^−^^3^
0 restraints	Δρ_min_ = −0.21 e Å^−^^3^

### Special details

**Table:**

Geometry. All esds (except the esd in the dihedral angle between two l.s. planes) are estimated using the full covariance matrix. The cell esds are taken into account individually in the estimation of esds in distances, angles and torsion angles; correlations between esds in cell parameters are only used when they are defined by crystal symmetry. An approximate (isotropic) treatment of cell esds is used for estimating esds involving l.s. planes.

### Fractional atomic coordinates and isotropic or equivalent isotropic displacement parameters (Å^2^)

**Table:**

	*x*	*y*	*z*	*U*_iso_*/*U*_eq_	
Cl1	0.94481 (3)	0.31466 (16)	0.62511 (5)	0.0747 (3)	
C1	0.70850 (12)	0.6448 (4)	0.44590 (15)	0.0465 (4)	
H1	0.6623	0.7651	0.4608	0.056*	
C2	0.70013 (10)	0.5327 (4)	0.33486 (14)	0.0396 (4)	
C3	0.61893 (11)	0.6044 (4)	0.24400 (15)	0.0440 (4)	
H3	0.5806	0.7576	0.2617	0.053*	
C4	0.59348 (11)	0.4758 (4)	0.13885 (14)	0.0428 (4)	
H4	0.6301	0.3187	0.1196	0.051*	
C5	0.51318 (11)	0.5638 (4)	0.05308 (14)	0.0442 (4)	
H5	0.4768	0.7210	0.0727	0.053*	
C6	0.78361 (12)	0.5819 (4)	0.53446 (15)	0.0491 (5)	
H6	0.7879	0.6593	0.6081	0.059*	
C7	0.84667 (12)	0.2943 (5)	0.40343 (16)	0.0512 (5)	
H7	0.8936	0.1769	0.3892	0.061*	
C8	0.77155 (11)	0.3594 (4)	0.31545 (15)	0.0462 (4)	
H8	0.7685	0.2863	0.2417	0.055*	
C9	0.85169 (11)	0.4047 (4)	0.51293 (15)	0.0473 (4)	

### Atomic displacement parameters (Å^2^)

**Table:**

	*U*^11^	*U*^22^	*U*^33^	*U*^12^	*U*^13^	*U*^23^
Cl1	0.0578 (3)	0.0907 (5)	0.0617 (4)	0.0025 (3)	−0.0072 (2)	0.0067 (3)
C1	0.0474 (10)	0.0494 (10)	0.0469 (10)	0.0036 (8)	0.0197 (8)	−0.0014 (9)
C2	0.0397 (8)	0.0400 (9)	0.0410 (9)	−0.0025 (7)	0.0141 (7)	0.0023 (8)
C3	0.0416 (9)	0.0438 (10)	0.0492 (10)	0.0039 (7)	0.0166 (8)	0.0027 (8)
C4	0.0401 (8)	0.0418 (9)	0.0475 (10)	0.0008 (7)	0.0137 (7)	0.0052 (8)
C5	0.0399 (9)	0.0445 (10)	0.0492 (9)	0.0010 (7)	0.0135 (7)	0.0071 (8)
C6	0.0569 (11)	0.0522 (11)	0.0392 (9)	−0.0052 (9)	0.0145 (8)	−0.0021 (8)
C7	0.0406 (9)	0.0593 (12)	0.0542 (11)	0.0056 (8)	0.0137 (8)	0.0018 (10)
C8	0.0458 (9)	0.0535 (11)	0.0408 (9)	0.0003 (8)	0.0144 (7)	−0.0047 (8)
C9	0.0432 (9)	0.0486 (10)	0.0462 (10)	−0.0058 (8)	0.0054 (7)	0.0060 (9)

### Geometric parameters (Å, º)

**Table:**

Cl1—C9	1.7376 (17)	C4—H4	0.9300
C1—C6	1.381 (2)	C5—C5^i^	1.342 (3)
C1—C2	1.392 (2)	C5—H5	0.9300
C1—H1	0.9300	C6—C9	1.369 (2)
C2—C8	1.394 (2)	C6—H6	0.9300
C2—C3	1.464 (2)	C7—C8	1.379 (2)
C3—C4	1.333 (2)	C7—C9	1.382 (3)
C3—H3	0.9300	C7—H7	0.9300
C4—C5	1.439 (2)	C8—H8	0.9300
			
C6—C1—C2	121.66 (16)	C4—C5—H5	117.5
C6—C1—H1	119.2	C9—C6—C1	119.39 (17)
C2—C1—H1	119.2	C9—C6—H6	120.3
C1—C2—C8	117.41 (15)	C1—C6—H6	120.3
C1—C2—C3	119.58 (15)	C8—C7—C9	119.45 (17)
C8—C2—C3	122.99 (15)	C8—C7—H7	120.3
C4—C3—C2	127.62 (16)	C9—C7—H7	120.3
C4—C3—H3	116.2	C7—C8—C2	121.33 (17)
C2—C3—H3	116.2	C7—C8—H8	119.3
C3—C4—C5	124.43 (17)	C2—C8—H8	119.3
C3—C4—H4	117.8	C6—C9—C7	120.72 (17)
C5—C4—H4	117.8	C6—C9—Cl1	119.46 (15)
C5^i^—C5—C4	125.0 (2)	C7—C9—Cl1	119.81 (14)
C5^i^—C5—H5	117.5		
			
C6—C1—C2—C8	−1.4 (3)	C9—C7—C8—C2	−0.5 (3)
C6—C1—C2—C3	179.69 (16)	C1—C2—C8—C7	1.6 (3)
C1—C2—C3—C4	−168.49 (17)	C3—C2—C8—C7	−179.46 (16)
C8—C2—C3—C4	12.6 (3)	C1—C6—C9—C7	1.3 (3)
C2—C3—C4—C5	−178.71 (16)	C1—C6—C9—Cl1	−178.05 (13)
C3—C4—C5—C5^i^	180.0 (2)	C8—C7—C9—C6	−1.0 (3)
C2—C1—C6—C9	−0.1 (3)	C8—C7—C9—Cl1	178.31 (14)

Symmetry code: (i) −*x*+1, −*y*+1, −*z*.
